# Comparative Analysis of Total Cost of Ownership: Commercial-Grade versus Diagnostic-Grade Displays in Remote Radiology Workstations

**DOI:** 10.1007/s10278-025-01651-y

**Published:** 2025-09-08

**Authors:** Katie Hulme, Jennifer Arnold, Ryan Thomas, Roy Kittelberger, Namita Gandhi, Douglas Nachand, Po-Hao Chen

**Affiliations:** 1 Medical Physics, Diagnostics Institute, Cleveland Clinic, Cleveland, OH USA; 2 Imaging Informatics, Diagnostics Institute, Cleveland Clinic, Cleveland, OH USA

**Keywords:** Displays, Quality control, Cost of ownership, Remote radiology, DICOM-GSDF

## Abstract

With the increasing shift towards remote radiology work, institutions face the challenge of balancing cost-effectiveness with operational reliability. This experiential report presents a comparative analysis of the total cost of ownership (TCO) of commercial-grade displays (WCDs) and diagnostic-grade displays (WDDs) in remote diagnostic stations. We evaluate direct and indirect costs associated with each display type using activity-based costing, focusing on deployment, quality control (QC) processes, and ongoing maintenance. Our findings suggest that while CGDs offer significant initial cost savings, their long-term maintenance requirements, particularly in manual QC, increase recurring expenses. The crossover point for CGDs with monthly QC occurs at approximately five years against mid-level DGDs, potentially making DGDs a more sustainable option over time.

## Background

In recent years, a growing shift toward remote radiology workstations has spurred many radiology departments to adopt non-medical monitors for interpretive use [1–3]. However, complexities with display calibration and reading environments can complicate adherence to standards in remote settings, with offsite setups often requiring additional planning and resources to effectively implement the professional guidelines established by medical physics and radiology organizations [4–6].

Requirements for diagnostic displays used in radiology are limited to a few state/city-specific regulations and the Mammography Quality Standards Act (MQSA), which only addresses displays used for the interpretation of mammograms. This study focuses specifically on non-mammography displays, for which the lack of regulatory and accreditation requirements has resulted in varying implementations and degrees of quality control between practices. While some practices may still have limited or no quality control in place for non-mammography diagnostic displays, this “set and forget” approach is not endorsed by the American College of Radiology (ACR) or the American Association of Physicists in Medicine (AAPM), nor is it supported by research, which documents the importance of consistent image presentation [7, 8] and appropriate reading environments [9, 10]. This paper helps quantify the efforts needed to maintain adherence to the American College of Radiology (ACR) technical standards [5, 6] for diagnostic displays utilized in the remote environment.

Medical “diagnostic-grade” displays are engineered to meet stringent standards for accurate image presentation, consistency, and reliability. They generally feature higher brightness levels and luminance ratios, along with features designed to minimize glare and maintain uniform brightness across the screen. Diagnostic-grade displays commonly incorporate built-in photometers or colorimeters for automatically calibrating displays to the Digital Imaging and Communications in Medicine (DICOM) Grayscale Standard Display Function (GSDF), which is essential for ensuring the consistent and accurate presentation of medical images [4], as well as illuminance meters for monitoring ambient light. These technological features, along with robust warranties, are reflected in their high price point.

High-quality displays used for general computing purposes, in this manuscript termed “commercial-grade displays,” present a potentially cost-effective alternative to diagnostic-grade displays for non-mammography interpretation. While displays used for mammography are now required to meet applicable FDA premarket authorization requirements [11], there is no such requirement for other modalities. The lower initial cost of commercial-grade displays can make them attractive for practices that need to expand remote capabilities quickly and affordably. However, because these displays are not inherently designed for medical imaging purposes, care must be taken to select a display with appropriate specifications, and additional manual quality control processes and calibration are usually necessary to meet the recommendations of the applicable American College of Radiology (ACR) technical standards [5, 6]. Users additionally assume responsibility for establishing and implementing the applicable settings and performance criteria required to ensure backlight stability, luminance ratio, and GSDF conformance for the selected display, all of which are generally provided by the manufacturer for diagnostic-grade displays.

Early studies comparing diagnostic-grade and commercial-grade displays reported statistically significant performance differences, primarily attributed to the lower maximal luminance values and inadequate pixel density of commercial-grade color LCDs available at the time [12, 13]. Other work showed no significant diagnostic difference between the two types, as long as both are calibrated in accordance with DICOM GSDF [14]. Our prior work suggests evolving display panel technology means today’s monitors offer pixel pitches and luminance levels on par with or approaching those of some diagnostic-grade displays. When properly calibrated according to DICOM standards, some commercial-grade displays can also sustain adequate technical performance over time [15, 16].

These modern commercial-grade displays are seeing increased real-world use in the radiology environment. During the COVID-19 pandemic, some radiology practices successfully deployed home workstations using readily available commercial displays [2]. A UK survey of 217 radiology practices found that, 42% of the practices deployed home workstations with monitors that did not have self-calibration capabilities during the pandemic [1]. In these deployment scenarios, however, the indirect maintenance and support efforts necessary to maintain GSDF compliance for commercial-grade hardware may erode initial savings from lower direct equipment costs [15].

Unfortunately, a paucity of literature exists analyzing the cost and tradeoffs made when making radiology workstation procurement decisions. The diagnostic monitor is often the most costly component of the radiology workstation [17, 18], and scaling on-premise solutions to every individual radiologist’s home office in a large practice can be costly.

Procurement decisions for expensive equipment frequently prioritize initial acquisition cost, with a total cost of ownership (TCO) overlooked due to the complexity of accurately assessing long-term financial implications [19]. Focusing solely on initial costs may not account for recurring expenses such as maintenance, calibration, QC procedures, and equipment lifecycle. A TCO approach takes a long-term perspective and encompasses direct and indirect costs associated with the purchase, operation, maintenance, and eventual disposal of equipment over its lifecycle.

This study aimed to conduct a TCO analysis comparing a remote deployment of radiology workstations with commercial-grade displays (CGDs) and workstations with diagnostic-grade displays (DGDs), contrasting various setup options by varying the equipment selection and maintenance strategies. By combining direct equipment costs with activity-based costing (ABC) of downstream deployment and support, we present data from a multi-state, multi-center, large hybrid radiology practice estimating the initial cost for TCO for the procurement and implementation of a CGD versus a DGD, building the year-to-year upkeep cost of each workstation type, and performing a crossover point analysis to identify the point at which an initially cost-effective strategy may become more costly at a later time.

## Methods

### Institutional Pilot and Rollout of Remote Workstations

**Fig. 1:**
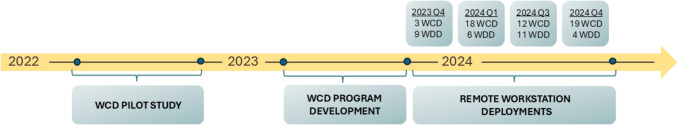
Timeline for institutional pilot and rollout of remote workstations. Initial experience deploying 52 workstations with commercial-grade displays (WCDs) was used to quantify indirect costs related to deployment, quality control, and technical support relative to workstations with diagnostic-grade displays (WDDs)

TCO analysis was informed by institutional experience and data collected from the deployment of 82 remote workstations over a 12-month period. A total of 52 WCDs and 30 WDDs were deployed between October 2023 and September 2024. Figure 1 outlines the scope and timeline of the project.

An initial pilot was conducted in 2022, prior to the rollout, with four WCDs utilizing a commercial-grade display (Dell Technologies, U3219Q) to assess suitability for a home radiologist workstation setup and to determine the frequency of manual display calibration that would be required of the radiologist should a similar setup be adopted at a larger scale. The display was calibrated to the GSDF and a white point of 350 cd/m^2^ using an external photometer (Barco, Inc., LCD sensor) and third-party software (Barco, Inc., MediCal QAWeb). The maximum luminance (*L*_max_) and maximum absolute deviation from GSDF were measured on a weekly basis and monitored over a 6-month period, with calibration performed on an as-needed basis when GSDF compliance was out of tolerance. Pilot data indicated that target *L*_max_ could be maintained within 5% and compliance with GSDF within 10% over this period; however, this required manual adjustments of overall display brightness and/or recalibration of the displays on several occasions due to backlight drift [20].

To simplify the QC workflow for end-users, it was determined that display calibration should not be performed on an as-needed basis, but rather on a routine basis, immediately followed by GSDF compliance testing. Pilot data indicated that a minimum of monthly calibration was likely warranted. The QC software for the deployed WCD solution permitted these two tests (calibration and compliance verification) to be executed in conjunction with each other under a single user-initiated QC task labeled “calibration.”

The decision to proceed with deploying WCDs to radiologists requesting home workstations for occasional remote shifts was made in early 2023. At this time, the consumer-grade display used in the initial pilot was no longer commercially available, and end-of-life had been announced for the QC software, with the next-generation platform (Barco, Inc., QAWeb Enterprise) lacking support for calibration of third-party displays. Therefore, a new display meeting the specifications of the 2022 ACR-AAPM-SIIM Technical Standard for Electronic Practice of Medical Imaging [5] was selected (Dell Technologies, G3223Q); the display was VESA DisplayHDR 600 certified with a 32-inch diagonal, 16:9 aspect ratio, and 0.1845 mm pixel pitch. Additionally, a new software platform (QUBYX, PerfectLum 4) and compatible handheld photometer (X-Rite, i1 Display Pro) were chosen for calibrating the diagnostic display in our WCD setup. Displays were calibrated to GSDF using an 18-point calibration, with the white point set to 375 cd/m^2^.

Displays dedicated to cardiovascular imaging and nuclear medicine were configured as a single screen. Most others, however, were configured to picture by picture (PBP) mode and thus were recognized by the QC software as two separate displays, with calibration and compliance testing having to be repeated for each half of the display. The decision to calibrate in PBP mode for these displays was made from the vantage point of minimizing the steps involved for the end-user when executing QC, as it was anticipated that asking the user to switch in and out of PBP mode would increase user error and be an additional hurdle to compliance. It is worth noting, however, that the implication of this decision was that different physical regions of the display were used to calibrate the two halves, thus non-uniformities in display luminance would introduce discrepancies in overall luminance between the two halves via the calibration process. This is not unlike having two side-by-side diagnostic displays, however, that are calibrated but not perfectly matched, which was a common setup prior to the introduction of large-format displays.

WCDs were deployed to radiologists with the handheld photometer used by the IT Analyst for initial calibration and verification testing. Radiologists were responsible for performing monthly quality control consisting of display calibration followed by GSDF compliance testing and visual assessment of a test pattern (TG-18QC).

Due to the anticipated scale of WCD deployment, significant effort was spent developing a deployment workflow, along with drafting detailed documentation for IT Analysts and radiologists, prior to deploying the first few stations. A spreadsheet was developed for IT Analysts to track both the completion of and time spent on each step of the deployment process. These timesheets were maintained for the deployment of the first 29 WCDs, and were later used to help quantify indirect costs affiliated with deploying WCDs relative to our standard WDD setup issued to full-time remote radiologists.

Fully remote radiologists were provided WDDs utilizing a diagnostic-grade 6MP display (Barco, Inc., MDCC-6530) registered to a quality control server (Barco, Inc., QAWeb Enterprise). Displays had a built-in photometer (Barco, Inc., iGuard), and performed automated compliance verification and calibration according to our institutional QC policy.

### Assessment of Direct Costs: Equipment

Total upfront equipment cost calculation accounted for each component of the workstation. A typical radiologist workstation consists of at least one display for diagnostic interpretation, 1–2 workflow displays, a computer with a high-quality graphics card and processor, a speech mic, and peripheral equipment such as a keyboard and mouse. A WCD will additionally require the purchase of a photometer and calibration software.

This analysis assumed a single wide-format display was being used for interpretation. Costs for this display were estimated from the average of various manufacturers’ suggested retail prices (MSRP), and were calculated for diagnostic-grade 6MPs, diagnostic-grade 8MPs, and commercially available displays meeting minimum recommended specifications outlined in the 2022 ACR-AAPM-SIIM Technical Standard for Electronic Practice of Medical Imaging [5].

Identical workstation builds, including graphics cards, were assumed for each of the three setups considered (WDD with 6MP, WDD with 8MP, and WCD). While there may be instances in which a diagnostic-grade display requires the use of a proprietary graphics card, further adding to the cost of the workstation, this was not factored into the presented analysis. Two 23″ monitors were also included in the cost. Costs for these components were derived from the prices for our institutional setup.

### Assessment of Indirect Costs: Deployment

Deployment costs were estimated by breaking our institutional process down into 15 distinct cost activities. The IT Analyst logged the time spent on each of the 15 cost activities for a given WCD in the deployment timesheet. Timesheets were maintained for the first 29 WCD deployed. The average time (in hours) for each activity was calculated across all 29 data points and multiplied by the assumed hourly wage of the individual performing the activity to obtain the estimated activity cost. National estimates of mean hourly wages, as reported by the US Bureau of Labor Statistics [21], were assumed when calculating activity-based costs (Table 1).

**Table 1:** National estimates of mean hourly wages, as reported by the US Bureau of Labor Statistics, assumed when calculating activity-based costs

Occupation	Mean hourly wage
IT analyst	$53.27
Medical physicist	$104.18
Radiologist	$170.17

**Fig. 2:**
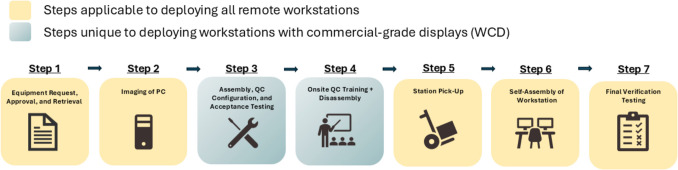
Flow diagram depicting our institutional deployment workflow. The deployment process was broken down into 15 distinct cost-activities, which were then categorized into seven general steps. Two of these steps were unique to the deployment of workstations with commercial-grade displays (WCDs). The average, min, and max time for each step was calculated from time sheets maintained by IT analysts during deployment of the first 29 WCDs to quantify indirect costs associated with deployment

Figure 2 summarizes our institutional deployment process for remote workstations, with the initial 15 cost-activities categorized down into seven general steps. These steps apply to deploying both WCD and WDD, except for the third and fourth steps, which are unique to deploying WCD.

The first step involves processing the initial request for a workstation, ensuring all prerequisites for receiving a workstation are met, and physically retrieving equipment from storage. Prerequisites are assessed by distributing a questionnaire to radiologists. The radiologist attests to each item in the questionnaire, including but not limited to provisions for internet bandwidth, wired internet connection, adequate desk space with sufficient weight capacity, and agreement to expectations regarding home workstation use (including QC, if applicable).

The workstation PC is imaged in step two. The standard image includes all software and applications routinely used in radiology. Any additional items to be installed may be indicated in the initial questionnaire. At the completion of this step, a workstation with a diagnostic-grade display is ready to be picked up or shipped directly to the radiologist (step 5). In many instances, particularly if the radiologist is located out of state, equipment for WDDs is shipped directly to the home of the radiologist. However, for the purposes of this analysis, on-site pick-up was assumed.

WCDs, on the other hand, undergo two additional steps prior to releasing the station. These stations are fully assembled, and the commercial-grade display to be used for interpretation undergoes an initial acceptance test, conducted by an IT analyst under the oversight of a medical physicist (step 3). Acceptance testing was informed by the recommendations of AAPM Report No. 270 [4] and consists of verifying the maximum and minimum luminance, ensuring the display is compliant with GSDF to within 10% across the luminance range, measuring display uniformity, and visually assessing a series of test patterns. All hand-held photometers were verified against three calibrated reference photometers (Raysafe, X2 Light Sensor) and confirmed to be within 10% of the average luminance value measured by the reference photometers at three grey levels spanning black to white.

Upon successful completion of acceptance testing, the radiologist is notified that their WCD is ready, and a time is coordinated for them to come on site to receive QC training and take their station home (steps 4 and 5). The station remains fully assembled until this date, as QC training involves a hands-on demonstration of how to calibrate their diagnostic display with their assigned photometer using the QC software and how to run the scheduled visual check of the test pattern. Detailed take-home instructions are provided, outlining the procedures reviewed during hands-on training. Once training is complete, the station is disassembled and boxed up. The IT analyst assists with loading equipment in the radiologist’s vehicle, but it is the responsibility of the radiologist to take the equipment home (step 5). Drive time between the radiologist’s home and pickup location was not factored into the cost analysis, as this was highly variable with each radiologist. Only the time spent on-site retrieving and loading equipment was included.

Once at home, the workstation is fully assembled by the radiologist, with remote assistance from the IT analyst, as needed (step 6). Detailed instructions for assembly are provided to all radiologists. Once assembled, a series of final checks are conducted (step 7). For WCD, radiologists are instructed to repeat the QC tests and confirm they are passing. For WCD and WDD, the IT analyst remotes into the station to verify connectivity, and ensures that manual or automated QC tests have transferred to the respective QC server and are in compliance.

It should be noted that some practices may choose to conduct physical in-person acceptance testing (step 3) of remote WDDs as well, either before or after deployment. However, the built-in tools and software for WDDs permit remote configuration and verification of several, but not all, metrics that would be assessed during acceptance testing. Thus, many practices rely on these tools to ensure compliance due to the logistical complexities of acceptance testing for remote stations. For WCD, however, this workflow cannot be considered, as the QC software licenses are coupled directly with the deployed PC, and the full setup must be verified to ensure all components are working together properly, and that the display is capable of GSDF compliance. Additionally, the commercial-grade displays are not engineered to meet the performance recommendations for display uniformity, brightness, etc.; thus verification that these metrics are met is essential as they are not guaranteed.

### Assessment of Indirect Costs: Quality Control and Technical Support

The QC histories for the 52 WCD units and 30 WDD units deployed over the 12-month period were extracted for analysis to quantify and compare longitudinal performance of the commercial-grade displays relative to the diagnostic-grade displays, and to determine typical failure rates in GSDF compliance (used to inform the assumptions used to estimate the activity-cost for monthly QC). QC histories were also extracted for 151 diagnostic-grade displays that had been in use for over 3 years. All data points were included for diagnostic-grade displays and commercial-grade displays operated in regular mode. For displays operated in PBP mode, only the results from the right half of the display were analyzed, to simplify the analysis and avoid weighting the results of these displays more heavily than their counterparts. Maximum absolute deviation from GSDF (|%ΔGSDF_max_|) and deviation from the target white point (%Δ*L*_max_) were determined for each data point; average and standard deviation, weighted by the number of data points per display, were then calculated for each metric as a function of display model. The rate of failure in GSDF compliance (defined as |%ΔGSDF_max_|> 10%) was additionally calculated.

Ongoing maintenance costs for remote workstations were broken down into four cost-activities. Approximate time spent on each activity over the course of a year was estimated on a per workstation basis, and the affiliated cost was derived from the assumed hourly wage of the responsible individual (Table 1):

1. General IT support and troubleshooting
2. User-initiated QC (WCDs only)
3. IT support and troubleshooting for QC (WCDs only)
4. Programmatic QC oversight (WCDs only)

All remote workstations, regardless of whether they are WCD or WDD, require general support. The IT analyst responds to calls submitted to the IT Help Desk, which may be related to network connectivity, hardware issues, or software access or functionality, and are responsible for ensuring required updates to the operating system and installed applications are scheduled and completed. The degree of support is highly variable with each workstation. A best-estimate of an average of 3 h per year per station spent on these tasks was provided by the informatics team responsible for supporting remote workstations. The same amount of time was assumed for WCD and WDD for general support.

The remaining activities are specific to supporting workstations with commercial-grade displays. Unlike WDDs, which can run scheduled QC tasks automatically without user intervention, WCDs require the use of an external photometer and user-initiation to accomplish these tasks. The cost affiliated with manual QC will depend on the frequency at which QC is performed, as well as the rate of failure. For the purposes of this analysis, it was assumed that QC is performed monthly with a 20% failure rate in GSDF compliance, in which instance QC is repeated a second time to recalibrate the display. Failure rate was derived from the analysis of QC test results collected over the deployment lifetimes of the 52 WCDs. Our institutional QC procedure, consisting of display calibration followed by GSDF compliance testing and visual assessment of a test pattern, was timed from start to finish and took approximately 10 min. Using these assumptions, it was estimated that the radiologist spends 144 min, on average, performing QC each year.

If failures in GSDF compliance could not be resolved with subsequent recalibrations, the expectation from administration was that the radiologist would come on site to read until the issue was resolved or their WCD was replaced. While this scenario is anticipated to occur, there were no instances of this occurring within the study period that could be used to inform an estimate of the frequency at which it could be expected. This scenario is therefore not accounted for in this analysis.

Calls received by the Radiology IT Help Desk that are specific to WCD QC are escalated to WCD subject matter experts (SMEs), a subset of the IT analysts that support remote workstations. These calls may be related to repeated failures in display calibration, or issues with the QC software itself. Again, the degree of support is highly variable with each workstation, and not all issues are captured by the IT Help Desk, as many radiologists reach out directly to the SME for assistance. Given our preliminary experience, a best-estimate of 30 min per workstation per year spent troubleshooting QC was assumed for both the radiologist and the IT analyst. Lastly, a WCD quality assurance team consisting of IT Analyst SMEs, a Medical Physicist, and the Radiology Chief Informatics Officer was established to develop the processes and documentation necessary to support the large-scale deployment of WCDs. This team was responsible for oversight of the QC program and was additionally tasked with identifying and following up on non-compliances, such as failure to perform QC or unaddressed QC failures. The WCD QA team met for 1 h per week for the first 6 months of the WCD rollout, but the frequency has since been reduced to 1–2 meetings per month as needed, with follow-up items pertaining to programmatic oversight being delegated to the medical physicist. For the purposes of this analysis, it was assumed that the medical physicist spends 30 min per workstation per year on programmatic QC oversight for WCDs. This corresponds with an average of 30 min per week for 52 total workstations, consistent with our experience 6 months into an established program.

WDDs also require monitoring and support for automated QC. Issues with automated QC can generally be resolved remotely, with Help Desk assistance from the manufacturer when needed. Time spent by IT analysts addressing and resolving these issues is not always captured via the internal IT Help Desk, however, as these requests are not generally initiated by the end user. While these stations do require oversight, they have not necessitated the degree of ongoing programmatic investment demanded by the WCD. For the purposes of this analysis, it was assumed that these tasks were covered by the time estimate for general IT support and troubleshooting.

### Assessment of Total Cost of Ownership

Total cost of ownership was estimated using a cash flow analysis. The capital expenditure for each workstation configuration (WDD with 6MP, WDD with 8MP, and WCD) was determined by aggregating the costs of equipment procurement and deployment to estimate the initial acquisition cost. Recurring operational expenditures associated with ongoing quality control and technical support were quantified as a yearly expense and discounted to their net present value (NPV) using an assumed inflation rate of 2.9% [22] and projected over the lifespan of the workstation. A terminal value was not added to the NPV calculation as the workstation’s lifecycle and all components of the workstation, including the display, have an expected lifespan shorter than the projection timeline.

TCO estimates in this study did not account for monitor replacement, although it is anticipated that the lifetime of the selected commercial-grade display will not extend to the 5 years assumed. It is worth noting that one of the 52 WCDs thus far had failures which could not be resolved with IT support. Due to the involved nature of configuring the QC policy and acceptance testing, a completely new station was built and deployed to replace the WCD with the failing diagnostic display. Warranties for consumer-grade displays, unlike those for diagnostic-grade displays, do not cover GSDF compliance and may not guarantee the desired white point for calibration over the lifetime of the display. Thus, failures in GSDF compliance or white point that cannot be resolved with subsequent display calibration will require the WCD display to be replaced, incurring additional cost. Diagnostic-grade displays, on the other hand, will generally guarantee these items over a set time frame (i.e., 5 years) or backlight-hour run time.

Only costs directly related to individual workstations were considered in this analysis. Programmatic costs affiliated with initially establishing the rollout of WCD, such as the server setup, initial process development, and training of SMEs, were not accounted for in TCO estimates, though these costs were also non-negligible.

## Results

### Direct Costs: Equipment

Total equipment cost for each workstation configuration was $16,531, $10,512, and $7365 for the WDD with 6MP, WDD with 8MP, and WCD, respectively (Table 2), demonstrating that use of a commercial-grade display can reduce the upfront equipment cost of a workstation by ~ 55% relative to a standard diagnostic-grade 6MP display.

**Table 2:** Listed equipment costs were based on the average of various manufacturers’ suggested retail prices (MSRP), where applicable. Identical workstation builds (including graphics card) were assumed for all setups. Workstations with commercial-grade displays require the additional purchase of a photometer and third-party software for calibrating the display to the DICOM Grayscale Standard Display Function (GSDF), whereas diagnostic-grade displays generally have built-in photometers, and the cost of the photometer and calibration software is included in the cost of the monitor

DIRECT COSTS:	Commercial grade	Diagnostic grade	
		“High-end” 6MP	“Mid-level” 8MP
	*MSRP*_av_	*MSRP*_*av*_	*MSRP*_*av*_
**Workstation** Tower, graphics card, processor, etc	$3200	$3200	$3200
**Peripheral equipment** List displays, speech mic, networking solutions, etc	$1730	$1730	$1730
**Diagnostic display**	***$1155***	***$11,601***	***$5582***
**QC software license + photometer**	$1280	–	–
	**$7365**	**$16,531**	**$10,512**

The cost of the display itself accounts for up to 50–70% of the total equipment cost for workstations with diagnostic-grade displays. The increased cost relative to commercial-grade displays is due to the inclusion of features such as built-in photometers, software for automated QC, backlight stabilization, uniformity luminance correction, and integrated display controllers.

Licenses for QC software, and access to a local or cloud-based server that permits remote monitoring of QC results, are also generally included in the cost of a diagnostic-grade display. For WCD, however, these must be factored in as an additional upfront cost, alongside the purchase of a compatible photometer.

Diagnostic-grade 6MP displays have been available for several years from manufacturers of medical displays. Diagnostic-grade 8MP displays have only become available more recently, as existing 4 or 5 K panels have been adapted for medical use. These 8MP displays often have less powerful backlights relative to 6MPs, partially accounting for their lower price point.

### Indirect Costs: Deployment

Average time spent on each step of the deployment process, derived from time sheets maintained during the deployment of the first 29 WCDs, and affiliated activity-costs are presented in Table 3. WCDs necessitated stations to be built in full for the purposes of calibration, acceptance testing, and radiologist QC training, and then disassembled for distribution. Therefore, overall deployment activities were estimated to add an additional $784, on average, to the TCO for WCD, compared to $348 for WDDs.

**Table 3:** Average time spent on each deployment step, the responsible party, and the estimated cost. Time estimates were derived from timesheets maintained by IT analysts

Indirect costs: deployment (per workstation)		Commercial-grade		Diagnostic-grade	
	Party	Av. time (min)	Estimated cost	Av. time (min)	Estimated cost
**Equipment request, approval, retrieval**	IT analyst	106	$94	106	$94
**Imaging of PC**	IT analyst	83	$74	83	$74
**Assembly, QC config, acceptance testing**	IT analyst	145	$129	0	$ -
**Onsite QC training + disassembly**	IT analyst	50	$44	0	$ -
	Radiologist	50	$142	0	$ -
**Station pick-up**	IT analyst	37	$33	12	$11
	Radiologist	37	$104	12	$34
**Self-assembly of workstation**	Radiologist	50	$142	40	$113
**Final verification testing**	IT analyst	25	$22	25	$22
			**$784**		**$348**

### Indirect Costs: Quality Control and Technical Support

**Fig. 3:**
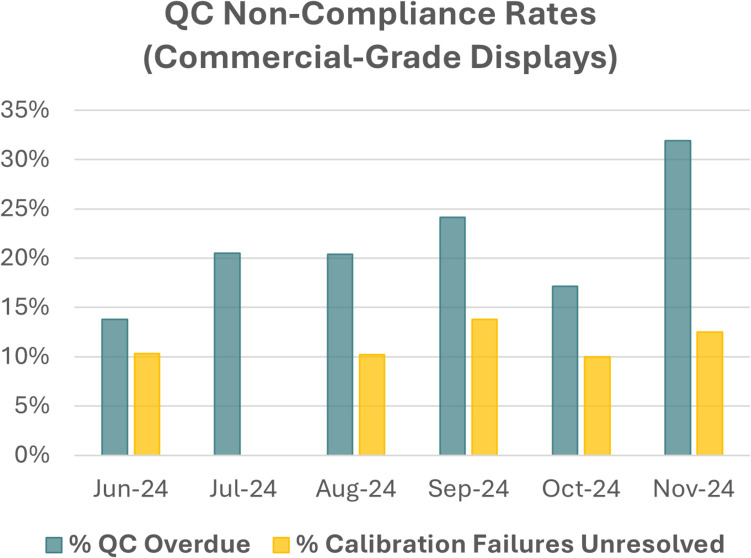
While diagnostic-grade displays generally have built-in photometers and can automatically run scheduled QC tasks, calibration of commercial-grade displays requires user-initiation. Compliance tracking was initiated in June 2024. Reports were generated from the QC server to flag workstations for which monthly QC was overdue (defined as not having been performed in > 45 days) and/or the most recent calibration had resulted in a failing result and had been left unresolved

While diagnostic-grade displays generally have built-in photometers and can automatically run scheduled QC tasks, calibration of commercial-grade displays requires user-initiation. Compliance tracking was initiated in June 2024. Reports were generated from the QC server to flag workstations for which monthly QC was overdue (defined as not having been performed in > 45 days) and/or the most recent calibration had resulted in a failing result and had been left unresolved, and results are summarized in Fig. 3. The number of stations flagged for user-initiated QC not being performed increased from 14 to 32% over 6 months, as both the number of deployed stations and length of deployment increased. In 10–15% of cases, failures encountered during routine QC were not identified and remedied with subsequent calibration by the user.

Few benchmarks exist within the literature with which to compare these rates, however, these values were considered unacceptably high. Frequency of non-compliances and time required to manually follow up with radiologists has necessitated process improvements, including improved documentation and communication, as well as implementation of automated emails (with assistance from in-house developers). The success of these additional measures is yet to be determined.

Analysis of QC records for newly deployed and existing workstations is presented in Table 4. Conformance with DICOM GSDF was achieved with the commercial-grade display, however, the average absolute maximum deviation from GSDF (|%ΔGSDF_max_|) was 10.8% with a failure rate of 20%, compared to 3.4% and a failure rate of 1.5% for the recently deployed diagnostic-grade displays. The backlights for the commercial-grade display exhibited greater drift than diagnostic-grade; the average %Δ*L*_max_ was − 3.56% (SD 4.92%), compared to %Δ*L*_max_ =  − 0.01% (SD 0.03%) for the diagnostic-grade displays.

**Table 4:** QC histories for 52 commercial-grade displays and 30 diagnostic-grade displays deployed within a 12-month period were extracted and analyzed, as were the histories for 151 diagnostic-grade displays that have been in use for several years. Maximum absolute deviation from GSDF (|%ΔGSDF_max_|) and deviation from the target white point (%Δ*L*_max_) were determined for each data point; average and standard deviation, weighted by the number of data points per display, were then calculated for each metric as a function of display model. Failure rate reflects the percentage of instances in which the (|%ΔGSDF_max_|) measured during compliance testing exceeded 10%

		Display model	#	Usage		GDSF compliance			
				Av. Data range	Backlight runtime *(average)*	Av. # Data points	Failure rate *(wt. average)*	Max % deviation *(wt. average)*	STD *(wt. average)*
				(Days)	(h)	#	%	|%ΔGSDF_max_|	STD (|%ΔGSDF_max_|)
Newly deployed (< 12 months)	**Commercial-Grade **	Dell G3223Q	52	128	*	16	20.0%	10.8%	12.8%
Newly deployed (< 12 months)	Diagnostic-Grade	Barco MDCC-6530	30	238	3534	34	1.5%	3.4%	3.6%
3+ years old	**Diagnostic-Grade**	Barco MDCC-6130	7	4267	26,611	520	1.9%	4.3%	2.50%
		Barco MDCC-6230	3	2796	27,849	247	10.8%	51.7%	168.3%
		Barco MDCC-6330	3	1957	31,057	220	0.3%	3.9%	9.5%
		Barco MDCC-6430	85	1920	17,742	217	0.4%	3.1%	3.9%
		Barco MDCC-6530	53	965	9093	108	0.8%	3.6%	4.2%
		Display model	#	White point					
				Av. # Data points	Target *L*_max_	Measured *L*_max_* (wt. average)*		Av. deviation from target *L*_max_* (wt. average)*	STD (%∆*L*_max_)* (wt. average)*
				#	(cd/m^2^)	(cd/m^2^)		%∆*L*_max_	STD (%∆*L*_max_)
Newly deployed (< 12months)	**Commercial-grade**	Dell G3223Q	52	16	375	361.7		− 3.56%	4.92%
Newly deployed (<12 months)	**Diagnostic-grade**	Barco MDCC-6530	30	34	600	599.9		− 0.01%	0.03%
3+ years old	**Diagnostic-grade**	Barco MDCC-6130	7	3259	400	400.2		0.05%	0.31%
		Barco MDCC-6230	3	1225	450	439.4		− 2.35%	10.98%
		Barco MDCC-6330	3	1228	450	449.9		− 0.20%	0.03%
		Barco MDCC-6430	85	1268	600	600.0		0.01%	0.04%
		Barco MDCC-6530	53	616	600	599.7		− 0.05%	0.08%

Backlight stability and GSDF conformance for the 151 diagnostic-grade displays that had been in use for several years were also superior to the newly deployed commercial-grade displays, even after significant backlight runtime. With the exception of one model (for which a single display had routine compliance issues at the end of its lifetime), these displays were able to maintain |%ΔGSDF_max_|< 5%, a failure rate < 2%, and %ΔL_max_ <  ± 1% for up to 12 years.

 The estimated activity-cost for yearly maintenance was $732 for WCDs, compared to $160 for WDDs (Table 5). Contributing factors for the WCD’s higher yearly activity cost were manual QC, additional IT support for QC failures and software issues, and physicist oversight related to QC. Of these, radiologist time spent performing QC was the largest driver, accounting for $408 of the $732. This estimate assumed a 20% failure rate for WCDs, as measured in Table 4, requiring the radiologist to repeat display calibration a second time.

**Table 5:** Activity-cost for yearly maintenance on a workstation with a commercial-grade display depends on the frequency of manual quality control (QC), frequency of QC failures, degree of difficulty in troubleshooting failures, and time required to track and enforce routine user-initiated QC. Time estimates in this table assume quality control is performed monthly with a 20% failure rate (requiring repeated display calibration)

Indirect costs: ongoing (annual) (per workstation per year)		Commercial-grade		Diagnostic-grade		
	Party	Av. time (min)	Estimated cost	Av. time (min)		Estimated cost
Quality control (manual)—monthly	Radiologist	144	$408	0	$ -	
IT support—troubleshooting—general	IT Analyst	180	$160	180	$160	
IT support—troubleshooting—WCD QC	IT Analyst	30	$27	0	$ -	
	Radiologist	30	$85	0	$ -	
QC oversight	MP	30	$52	0	$ -	
			**$732**		**$160**	

### Total Cost of Ownership

Use of a commercial-grade display reduces upfront expenses by approximately 48% compared to a WDD with a standard 6MP display, and 25% compared to a WDD with an 8MP display (Table 6). Year 1 cost for a workstation with a commercial-grade display was $8861, compared to $11,015 and $17,035 for a workstation with a mid-level 8MP and high-end 6MP, respectively, and was primarily driven by equipment cost.

**Table 6:** Total cost of ownership (TCO) per year of ownership was estimated using the initial acquisition cost and the yearly recurring cost discounted to the net present value (NPV) assuming an inflation rate of 2.9%

	**Commercial grade**		**Diagnostic grade**			
	***(QC—monthly)***		**“High-end” 6MP**		**“Mid-Level” 8MP**	
Initial equipment cost	$7365		$16,531		$10,512	
Initial deployment cost	$784		$348		$348	
**Total initial expenditure**		**$8149**		**$16,879**		**$10,860**
Yearly maintenance cost	$732.03		$159.81		$159.81	
Yearly maintenance cost—discounted to NPV		**Estimated TCO**		**Estimated TCO**		**Estimated TCO**
Year 1	$711	$8861	$155	$17,035	$155	$11,015
Year 2	$691	$9552	$151	$17,186	$151	$11,166
Year 3	$672	$10,224	$147	$17,332	$147	$11,312
Year 4	$653	$10,877	$143	$17,475	$143	$11,455
***Year 5***	$635	***$11,511***	$139	***$17,613***	$139	***$11,594***
Year 6	$617	$12,128	$135	$17,748	$135	$11,728
Year 7	$599	$12,727	$131	$17,879	$131	$11,859
Year 8	$582	$13,309	$127	$18,006	$127	$11,986
Year 9	$566	$13,875	$124	$18,130	$124	$12,110
Year 10	$550	$14,425	$120	$18,250	$120	$12,230

The TCO over the expected lifespan (assumed to be 5 years) for the workstation with the commercial-grade display ($11,511) was just about the same as that of the station with the mid-level 8MP display ($11,594) but less than that of the high-end 6MP display ($17,613).

**Fig. 4:**
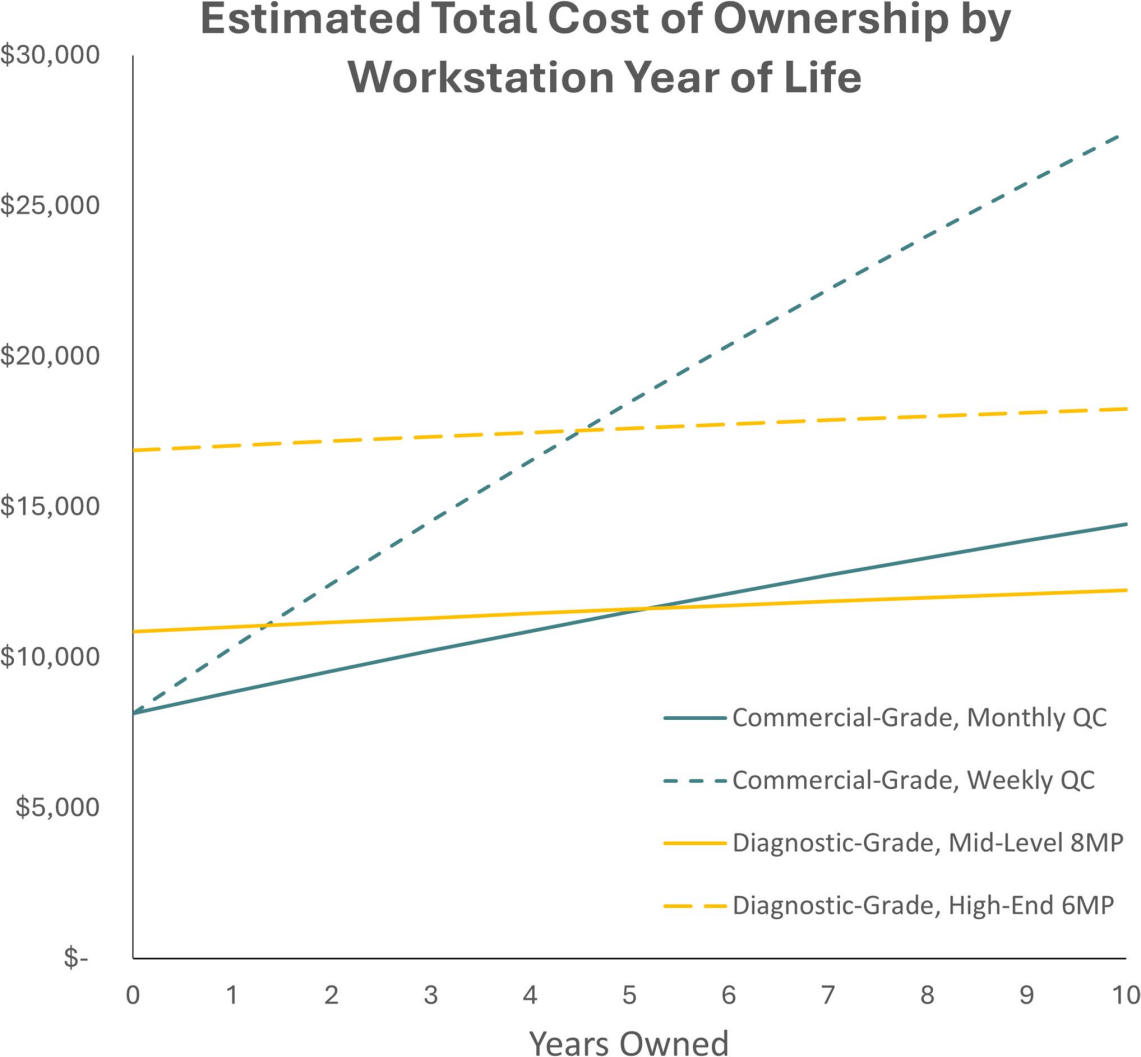
TCO projected over 10 years for average activity-cost estimates. Workstations with commercial-grade displays (WCD) have lower initial costs, but higher incremental yearly costs (sensitive to frequency of manual QC) relative to stations with automated QC. Crossover-point analysis indicates that TCO of workstations with diagnostic-grade displays (WDD) may equal that of a WCD after five years of ownership for some configurations, at which point the diagnostic-grade display will generally be under warranty

Crossover point analysis was conducted to identify when a WDD becomes more cost-effective than a WCD with routine quality control (Fig. 4). The results indicate that under the assumptions made, the WDD with the 8 MP display becomes cost-effective after 5.2 years when the QC for WCDs is performed monthly and significantly sooner, at 1.4 years, if QC is conducted weekly. The WDD with a 6MP display does not become cost-effective over the projected time frame (10 years) relative to a WCD with monthly QC, but reaches this point at 4.5 years if WCD QC is performed weekly. These findings highlight the impact of radiologist time performing QC on the long-term economic viability of various solutions.

## Discussion

The total cost of ownership (TCO) analysis reveals a balance between initial investment and long-term operational costs when selecting displays for remote radiology workstations. Workstations with commercial-grade displays (WCDs) offer a 25–50% reduction in initial expenses compared to workstations with diagnostic-grade displays (WDDs), appealing to institutions with budget constraints or a need for rapid remote capability expansion. However, WCDs entail hidden costs over time, requiring additional hardware and software for manual calibration, which increases deployment complexity and labor demands for IT personnel and radiologists while introducing variability in imaging compliance.

While efforts involved in program operations were quantified using the TCO framework, costs affiliated with program development were not captured. Program development encompasses the building of processes and documentation, as well as motivating the individuals involved in execution and equipping them with the tools and education they need to be successful. It also involves minimizing barriers to completion, whether that is time, frequency, or complexity, as these are all likely to reduce user compliance. The success of a quality control program for WCDs relies on the buy-in and engagement of every end-user, many of whom have competing priorities and limited bandwidth. This stands in contrast to WDDs, where oversight and follow-up can be consolidated into a select group of individuals. Rates of non-compliance observed in this study for WCDs indicate that despite substantial upfront efforts to train and educate, the investment required to obtain and maintain end-user engagement was underestimated. A practice selecting a WCD solution, if not accounting for such human factors, may be setting itself up for failure, regardless of cost.

This study contributes to the literature in three ways. First, commercial-grade displays have been generally believed to be feasible options for diagnostic use and have been deployed in real-life practice [1, 2]. The present study contributes quantitative data on the long-term precision and variability of commercial-grade displays with direct comparison to diagnostic-grade displays.

WCDs were found to have a significantly higher failure rate in maintaining DICOM GSDF compliance (20%) than WDDs (under 2%). It should be noted that GSDF compliance was measured immediately following display calibration for WCDs to streamline the QC workflow for radiologists. Had GSDF compliance been measured prior to calibration as well, it is very likely that the measured rate of failure would have been higher, as this would have more accurately captured backlight drift. However, it is still a relevant comparison to our WDDs, for which the calibration frequency had been adjusted from the manufacturer default (semiannual) to match the frequency of the compliance test (weekly). These results highlight the limitations of the WCD display in combination with the 18-point calibration in achieving tight GSDF compliance. Whereas the diagnostic-grade displays could routinely achieve GSDF compliance within 5%, the consumer-grade display averaged 10.8%. It is possible that tighter conformance could have been achieved with a higher-point calibration, however this option, if available, would have been prohibitively time intensive for routine manual QC. One of the primary advantages of the automated QC for WDDs is that it can be scheduled to run off-hours, thus execution time and frequency are decoupled from cost. This permits the use of more in-depth (“full”) calibration protocols, as well as the inclusion of color temperature calibration, which was not performed on our WCD displays, again, due to the extensive time required.

Secondly, the present study estimates the downstream impact of using these two display types in actual practice. The data presented demonstrate that maintaining calibration is costly and—in some cases—more costly than the workstation purchase itself. Variations in display performance that fall outside expected standards between diagnostic-grade and non-medical displays have previously been associated with reduced diagnostic accuracy and potential impact on care [12, 23]. However, previous research was typically conducted at a snapshot in time; the present results add to the literature that display performance is non-static, and routine calibration is both necessary and costly for commercial-grade displays. Across a WCD’s expected lifespan under a weekly QC strategy—necessary if the display performs inconsistently—the downstream indirect cost exceeds the initial expenditure. In contrast, diagnostic-grade displays have a higher initial equipment cost but appear to offset this disadvantage with reliability and automation of calibration, reducing the long-term costs, at least in high-utilization scenarios.

Finally, the study presents a nuanced recommendation about making purchase decisions: it depends on the intended use and longevity of the workstation. The TCO financial analysis suggests that the crossover point depends on both the initial equipment cost and the frequency of manual calibration, with WCDs with monthly QC becoming more costly compared to the proposed mid-level WDD specification at five years. Compared to the proposed high-level WDD specification, this cross-over point mathematically occurs at approximately 20 years. However, since this is substantially longer than the expected lifespan of a diagnostic workstation and the manufacturer-recommended lifecycle of both display types, the more appropriate conclusion is that WCD is likely more cost-effective than a high-level WDD in realistic usage. A weekly QC strategy for WCDs sharply increases the indirect cost over time, with the crossover point against a mid-level WDD occurring in the second year of ownership, and the crossover against a high-level WDD in the fifth year of ownership.

This constellation of results explains why WCD deployments at radiologist homes could have been a meaningful adjunct during the COVID-19 pandemic but may become more costly or challenging to maintain over time. Anecdotally, the authors’ institution began an at-home strategy by deploying WCDs for occasional home use where calibration frequency had less impact and was required either monthly or prior to use if usage frequency was less than monthly. For workstations with daily utility, such as those deployed on-premises or for remote-only radiologists, WDDs were the preferred deployment. The TCO analysis informed the authors’ institution to seriously consider mid-level WDDs for at-home use, despite the higher initial equipment cost.

## Limitations

Some limitations of this study are noted. First, although the direct cost data were derived from manufacturers’ recommended costs (MSRP), individual institutions may have their own negotiated rates that differ, which could substantially impact estimated crossover points. The proposed methodology, however, could be adapted by the reader to accommodate institution-specific equipment costs and derive their own respective crossover points for consideration. Although the study site is a multi-center, multi-state, mixed academic and community hospital environment, the estimated time collected from deployments may not generalize to all radiology practices. The salary data were derived using the US Bureau of Labor Statistics to mitigate risks of location-based biases. However, estimates made using the US BLS may not accurately reflect local wage variations or international contexts. Although our institution includes two international hospitals, radiology workstations in those facilities were not included due to substantial differences in equipment MSRP and wages.

Additionally, the study assumes consistent usage patterns and QC requirements over time, implying a linear increase in cost over time. Similarly, the study assumes stable inflation rates, typical in future-looking financial analyses but limited in predictive power. Advancements in display panel technology, changes in regulatory standards, shifts in clinical demand, and world-altering events could modify the cost dynamics, the expected lifespan of hardware, radiologist workflow, and inflation rates. Finally, variability in staff efficiency or unforeseen technical issues could result in different cost outcomes. The analysis also does not account for opportunity costs associated with efforts such as radiologists’ time spent on QC activities. Activity-based costing conventionally accounts for only the direct and indirect costs of contributing activities, whereas opportunity costs consider the value of the next-best activity rather than cost. Nevertheless, it is important to recognize that, by spending 144 min yearly on QC, radiologists would lose the opportunity to spend the same amount of time on an alternative activity such as revenue generation.

 Lastly, analysis of quality control records was limited to one model for the commercial-grade display, and five models from a single manufacturer for diagnostic-grade displays. Temporal backlight performance and GSDF failure rates may not be generalizable to other models, particularly if they employ substantially different display technology. Analysis was additionally informed by experience with a single QC software platform for WCDs.

Future directions include additional data collection on new workstations to validate findings in a broader range of clinical settings, including US and non-US radiology practices. Studies that directly assess the impact of display type on diagnostic accuracy and patient outcomes would provide insights into the clinical significance of these financial considerations. End-user usage patterns and survey feedback may be incorporated in future work to control for subjective differences in end-user perception.

## Conclusion

Commercial-grade displays may incur higher maintenance expenses over the long term compared to diagnostic-grade displays, which may be more costly upfront but offer more reliability and lower ongoing costs. The total cost of ownership analysis reveals an approximately 5-year crossover point between a workstation with a commercial-grade display compared to one with a mid-level diagnostic-grade display.
